# Branch-type intraductal papillary neoplasm of the bile duct treated with laparoscopic anatomical resection: a case report

**DOI:** 10.1186/s40792-020-00864-3

**Published:** 2020-05-15

**Authors:** Rumi Matono, Mizuki Ninomiya, Kazutoyo Morita, Takahiro Tomino, Yumi Oshiro, Tomoyuki Yokota, Takashi Nishizaki

**Affiliations:** 1 Department of Surgery, Matsuyama Red Cross Hospital, 1 Bunkyo-cho, Matsuyama City, Ehime 790-8524 Japan; 2 Department of Diagnostic Pathology, Matsuyama Red Cross Hospital, Matsuyama City, Ehime Japan; 3 Department of Center for Liver-Biliary-Pancreatic Disease, Matsuyama City, Ehime Japan

**Keywords:** Intraductal papillary neoplasm of the bile duct, Intraductal papillary mucinous neoplasm of the pancreas, Laparoscopic anatomical resection, Surgical margin, Segmentectomy

## Abstract

**Background:**

Intraductal papillary neoplasm of the bile duct (IPNB) is characterized by an intraluminal, growing papillary tumor covered by neoplastic biliary epithelial cells with a fine fibrovascular core. IPNB was introduced as a precancerous and early neoplastic lesion in the 2010 World Health Organization classification of tumors of the digestive system. IPNB eventually invades the bile duct wall and progresses to invasive cholangiocarcinoma. IPNB resembles intraductal papillary mucinous neoplasm of the pancreas (IPMN), particularly the main pancreatic duct type.

IPNB cases, possibly corresponding to branch-type IPMN, have been recently reported, and these cases involved the peribiliary glands significantly and showed gross cystic dilatation. Small branch-type intrahepatic IPNB often mimics simple liver cysts, making the diagnosis of IPNB difficult. Some literature recommended surgical resection for treatment. Laparoscopic resection is a good treatment option for small tumor. We herein present the case of branch-type IPNB that was treated with laparoscopic anatomical liver resection 5 years after being detected.

**Case presentation:**

A 64-year-old woman was undergoing follow-up for primary aldosteronism. In 2012, follow-up computed tomography (CT) incidentally revealed a 7-mm cystic lesion in segment 8 of the liver. From 2012 to 2017, the cystic lesion kept increasing in size, reaching 17 mm. In 2017, CT also revealed a 13-mm mural nodule in the cyst wall. Therefore, the patient was referred to our department for possible malignancy.

We suspected a branch-type IPNB; however, the mass was small and diagnosis could not be made without performing biopsy. Accordingly, surgical resection was performed for diagnosis and treatment. Because branch-type IPNB might show horizontal spread through the intrahepatic bile duct, we believed that anatomical resection of the liver was appropriate considering the malignant potential of the lesion. Therefore, laparoscopic anatomical resection of segment 8 of the liver was performed. The resected tumor measured 17 mm and was histologically diagnosed as a high-grade IPNB.

**Conclusion:**

Branch-type IPNBs are rare but can potentially lead to malignant tumors. Surgical resection is the treatment of choice, with laparoscopic anatomical resection being a good treatment option for this small tumor.

## Background

Intraductal papillary neoplasm of the bile duct (IPNB) is characterized by the presence of intraluminal papillary tumors with fibrovascular cores in the dilated bile ducts. IPNB was recently classified as a precancerous and early neoplastic lesion in the 2010 World Health Organization classification of tumors of the digestive system [1]. IPNB shares many clinicopathological features with intraductal papillary mucinous neoplasms of the pancreas (IPMN) [2–7]. IPNBs are often classified into 4 types, gastric, intestinal, pancreatobiliary, or oncocystic subtypes, similar to IPMNs and classified into 3 types based on location, intrahepatic, extrahepatic, and diffuse type [8–10]. IPNB arising from the large bile duct shares clinicopathological features with IPMN of the main pancreatic duct, including the high malignant potential and high frequency of the pancreatobiliary and intestinal types [8–12]. However, IPNB differs from pancreatic IPMN in several aspects. Most cases of IPNB are malignant, including carcinoma in situ, whereas IPMNs are often adenomas [2, 3]. Mucin secretion is higher in IPNB than in IPMN [3, 5, 6, 13]. IPMNs can involve the main pancreatic duct (main duct type), branch duct (branch type), or both ducts (combined type). The branch-type IPMN is usually multicystic and of the gastric subtype. IPNBs involving the extrahepatic bile ducts and intrahepatic large bile ducts may correspond to the main pancreatic duct type.

Recently, branch-type IPNB, the counterpart of branch-type IPMN, was reported [11]. Cystic micropapillary neoplasms were identified in the peribiliary glands, in branch-type IPNB [9, 11]. The peribiliary glands are accessory glandular tissues located around extrahepatic and major intrahepatic bile ducts [14, 15]. These accessory glands are distributed in the connective tissue surrounding the bile ducts or in the bile duct wall and connected to the bile duct lumen.

Small branch-type intrahepatic IPNB often mimics simple liver cysts, making the diagnosis of IPNB difficult. Most patients have been diagnosed after showing symptoms such as abdominal pain, fever, or jaundice [6, 7, 13]; however, some cases were asymptomatic and detected incidentally. When IPNB is detected, surgical resection is recommended to perform based on the malignant potential. Accordingly, IPNB cases develop from both the biliary epithelium and the peribiliary glands, with some cases invading the bile ducts; however, some cases were observed only in the cystic wall [2, 4, 5].

IPNB cells have a potential to spread along the bile duct epithelium [11, 16, 17]. In fact, the resection margin has a significant effect on the postoperative survival rate [17–26]. Operative managements depend on its location. For curative resection, resection of the involved liver with or without bile duct resection was performed [13, 15, 22–28]. Herein, we present the case of branch-type IPNB that was treated with laparoscopic anatomical liver resection in a 64-year-old woman.

## Case presentation

**Fig. 1:**
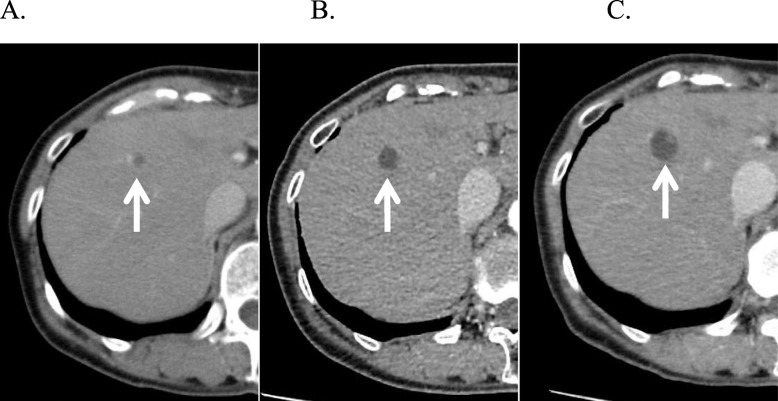
Computed tomography findings. Computed tomography scans showed **a** a 7-mm cystic nodule in 2012 (white arrow), **b** an 11-mm cystic nodule in 2016 (white arrow), and **c** a 17-mm cystic nodule in 2017 (white arrow) as well as a mural nodule in the wall

**Fig. 2:**
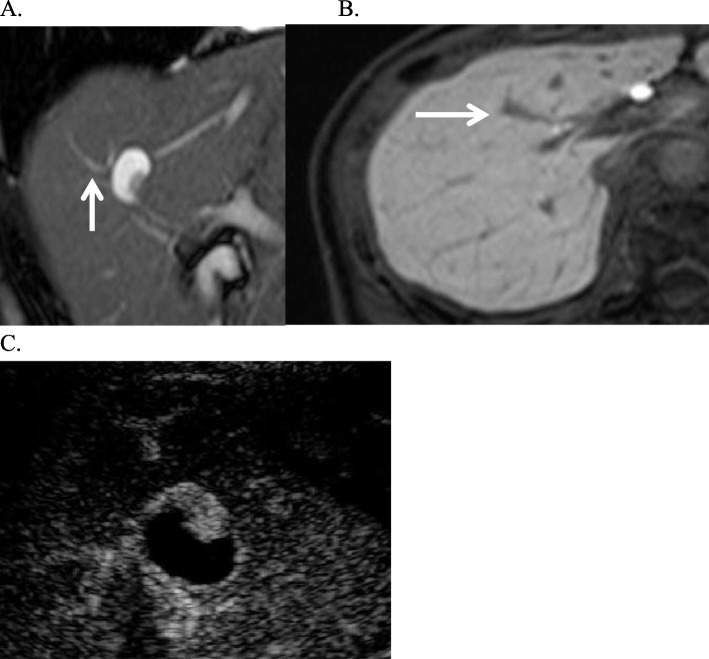
**a**, **b** Magnetic resonance imaging findings. **c** Ultrasonography findings. Magnetic resonance imaging showed slight dilation of the distal bile duct (white arrow). Ultrasound showed a highly echoic papillary nodular area in the cystic lesion

A 64-year-old woman was undergoing follow-up for primary aldosteronism at the Department of Internal Medicine of our hospital. The patient had hypertension, primary aldosteronism, and a goitrous tumor. The patient had last undergone follow-up computed tomography (CT) more than 10 years previously. However, in 2012, follow-up CT incidentally revealed a 7-mm cystic lesion in segment 8 of the liver. At that time, the cyst was considered benign and monitored regularly. Moreover, from 2012 to 2017, the cystic lesion kept increasing in size, reaching 17 mm. In 2017, CT also revealed a 13-mm mural nodule in the cyst wall (Fig. 1a–c); therefore, the patient was referred to our department for a possible malignant tumor. CT revealed that the proximal bile duct showed no abnormalities, such as dilation or stenosis, while the distal bile duct showed slight dilation, indicative of a mucin-producing biliary tumor. In addition, magnetic resonance imaging (T2-weighted imaging) revealed a 17-mm cystic lesion, with an internal signal intensity equivalent to that of water (Fig. 2a, b). Endoscopic ultrasonography revealed a 13-mm mural nodule in the cystic wall that was enhanced on contrast-enhanced ultrasonography (Fig. 2c). Laboratory measurements revealed normal level of liver enzymes. The carcinoembryonic antigen level was slightly elevated (5.3 ng/ml), but the levels of carbohydrate antigen 19-9, alpha-fetoprotein, and protein induced by vitamin K absence or antagonist-II were within the normal ranges.

We suspected a branch-type IPNB and thought that the presence of mural nodule suggested a potential of malignancy. However, the mass was small and diagnosis could not be made without performing biopsy. Several previous studies showed the usefulness of FDG-PET as a diagnostic tool to detect biliary malignancy [29]. However, considering the small tumor size, we considered CT, MRI, and contrast-enhanced ultrasonography were sufficient for the assessment of surgical indication. Thus, we have omitted FDG-PET from preoperative workup at this time.

**Fig. 3:**
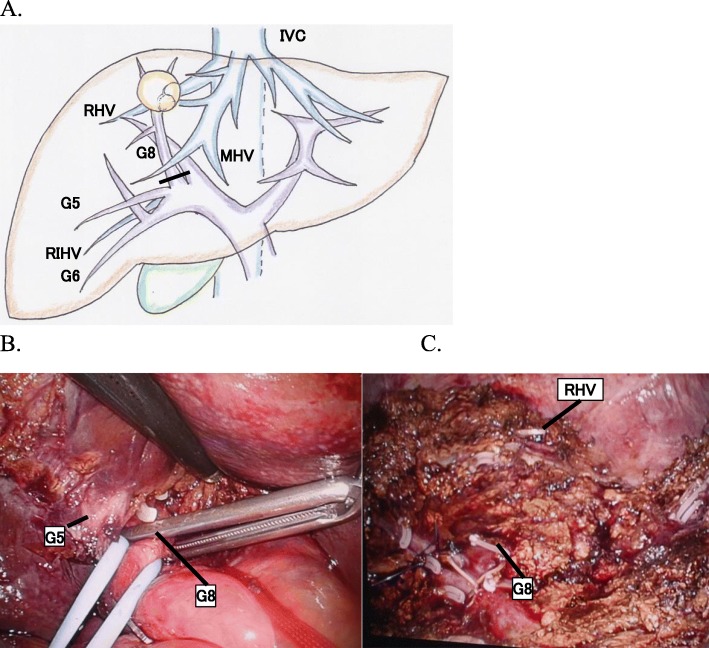
Anatomical observations and intraoperative findings. **a** Schema showing anatomy of the liver and the blood vessels. **b**, **c** Intraoperative views of the major steps of laparoscopic segmentectomy

Accordingly, surgical resection was planned for diagnosis and treatment. Because branch-type IPNB might show horizontal spread through the intrahepatic bile duct, we thought that anatomical resection of the liver was appropriate to obtain a sufficient surgical margin as much as possible. Accordingly, we performed laparoscopic anatomical resection of segment 8 of the liver via the extrahepatic Glissonean pedicle approach (Fig. 3a–c). After cystic plate cholecystectomy, the Glissonean pedicle of segment 8 was isolated and clamped by using a vascular clip (Fig. 3b). After confirming the ischemic demarcation line, the liver parenchyma was dissected along the marked line and the middle and right hepatic veins (Fig. 3c). During dissection, the Glissonean pedicle of segment 8 was ligated, clipped, and divided.

**Fig. 4:**
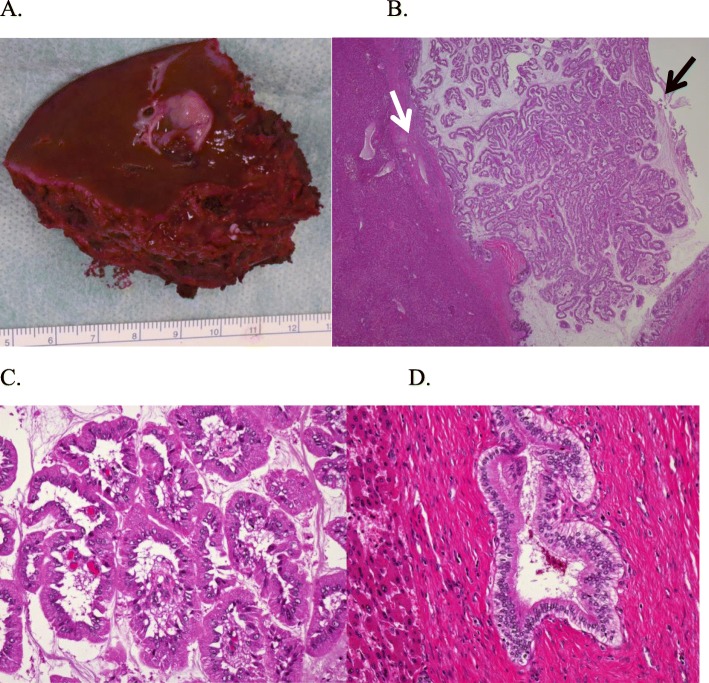
Macroscopic and microscopic findings of resected specimen. **a** Resected specimen showing a papillary tumor in the cystic wall. **b**–**d** Histopathological examination revealed the presence of an intraductal papillary neoplasm of the bile duct on hematoxylin and eosin (H&E) staining. **b** The papillary tumor cells (black arrow) can be observed in the cystic wall (white arrow) (H&E staining; magnification, × 40). **c** The tumor was a high-grade (H&E staining; magnification, × 400). **d** The tumor only located in the cystic wall and did not invade the wall of the adjacent bile duct (H&E staining; magnification, × 400)

Macroscopic examination revealed that the cyst was 17 mm and located in S8; the mural nodule was 13 mm (Fig. 4a) and showed mucin production. The bile ducts of segment 8 were visualized in addition to cystic space (Fig. 4a), suggesting that the cystic tumor communicated directly with the lumen of the adjacent bile duct. The tumor was very close to the bile duct. On microscopic histopathological examination, the resected specimen showed papillary growth and fibrovascular cores in the nodular area, comprising high-grade atypical epithelial cells in the cystic wall. However, papillary projection did not invade the adjacent bile duct lumen. The tumor was composed of papillary and glandular components; the origin of the present tumor might have derived from a peribiliary gland, not the large bile ducts.

Immunohistochemical analysis showed that the tumor was positive for MUC1, MUC5AC, MUC6, CK7, and CK20 and negative for MUC2. The final pathology was an IPNB, high grade (Fig. 4b–d) that did not invade the bile duct epithelium. The ductal margin at bile duct resection stump was also negative on histopathological examination.

The patient had an uneventful recovery, with no postoperative complications, and discharged 12 days after surgery. Follow-up abdominal CT after surgery did not reveal any mass, with the patient being asymptomatic 30 months after the surgery.

## Discussion

Recently, several case reports of branch-type IPNB were reported [2, 4, 5, 9, 15, 27, 28]. We summarized the characteristics of similar cases of branch-type IPNB (Table 1).

**Table 1:** Documented cases of branch-type IPNB

Author, reference number	Age (years)	Sex	Tumor site	Tumor size (mm)	Treatment
Fujita et al. [9]	70	Female	Cystic mass adjacent to the bile duct in segment 3	60	Left hemi-hepatectomy
Nakanishi et al. [15]	69	Female	Right anterior section	16	Right anterior sectionectomy
Tominaga et al. [27]	83	Female	Close to the bile duct in segment 2	25	Left hemi-hepatectomy
Hasebe et al. [28]	65	Male	Segment 8	33, 33	S8 segmentectomy
Present case	64	Female	Segment 8	17	Laparoscopic S8 segmentectomy

The long-term outcomes of surgically resected IPNB have recently been reported; Kim et al. reported the 5-year survival rate was 80.9%, with low proportion (9.3%) of positive resection margin [10]. The tumor location and size were important factors that influenced survival rate. Surgical margin affected their prognosis, and R0 resection should be done [7, 9, 15, 27]. Zhang et al. [26] reported additional resection of a positive frozen ductal margin to achieve R0 resection was associated with improved long-term outcomes. Although we were aware of such importance of resection margin status, we did not perform intraoperative diagnosis of bile duct margin because of the following 2 reasons. Firstly, if intraoperative diagnosis of bile duct margin was positive, it was thought that right hemi-hepatectomy was necessary to obtain sufficient additional margin. However, the expected remnant volume of the left liver was not sufficient enough in the current case. Therefore, we thought that two-stage hepatectomy would be a better choice in case of positive bile duct margin. Secondly, taking into account the small tumor size and preoperative images, we thought that possible positive margin status would be carcinoma in situ rather than invasive carcinoma. Previous literatures revealed that the prognosis of the patients with carcinoma in situ at the bile duct margin was significantly better than that with residual invasive carcinoma [22–26]. Furthermore, they also reported discrimination between severe dysplasia and carcinoma in situ at the surgical margin was difficult and sometimes frozen margin negative turned positive in final diagnosis. Thus, we decided not to perform intraoperative diagnosis of bile duct margin.

Fujita et al. [9] reported a case of branch-type IPNB, in which cystic mass located adjacent to the bile duct in segment 3 and they performed left hepatectomy. Nakanishi et al. [15] reported a case of the similar tumor, in which cystic mass located anterior section and they performed anterior sectionectomy. The cystic mass showed direct communication with the surrounding bile ducts, but the resection margin of the bile duct resulted in negative. Tominaga et al. [27] reported cystic mass located close to the bile duct in segment 2, and they performed left hepatectomy. Hasebe et al. [28] reported two cystic tumors located anterior and posterior bile duct in segment 8, and they performed segment 8 segmentectomy. All these cases were resected via open hepatectomy. However, these literatures did not mention whether intraoperative frozen-section analysis was performed.

The current case demonstrated the following characteristics: (1) the cystic tumor was incidentally detected on CT, and therefore, its size was small; (2) the cystic lesion was located within the periductal connective tissue around the periductal intrahepatic bile duct; and (3) the direct luminal communication was suspected between the cyst and the bile duct, because of the dilatation of the distal bile duct. Regarding the selection of the surgical procedure, there is, due to its rarity, no clear evidence to date supporting the superiority of performing anatomical resection over partial resection in the case of branch-type IPNB. However, considering the possibility of horizontal spread through the intrahepatic bile duct, anatomical resection, not partial resection, would be appropriate to obtain sufficient surgical margin as much as possible. Another reason to select anatomical resection is that because the branch-type IPNB arises from peribiliary glands around extrahepatic or major intrahepatic bile ducts, it usually locates relatively deep parenchyma of the liver as in the current case, but rarely around the surface of the liver [14]. Laparoscopic hepatectomy is safe, effective, and minimally invasive [16, 17]. The current case was a very small tumor; therefore, laparoscopic resection was applicable, and in case suspecting invasion of the epithelium of the bile ducts, anatomical segmentectomy would be appropriate. Moreover, we considered the current case to be feasible to re-perform right hemi-hepatectomy in case positive margin was detected in the final pathological diagnosis.

## Conclusion

In summary, branch-type IPNBs are rare but can potentially lead to malignant tumors; they might also horizontally spread through the intrahepatic bile duct. The choice of laparoscopic anatomical resection can be a good treatment option even for small tumors, as observed in the current case.

## Data Availability

All data generated during this study are included in this published article.

## Abbreviations

IPNB: Intraductal papillary neoplasm of the bile duct
IPMN: Intraductal papillary mucinous neoplasm of the pancreas
CT: Computed tomography
